# Evaluation of the National Tuberculosis Surveillance System in Sana’a, Yemen, 2018: Observational Study

**DOI:** 10.2196/27626

**Published:** 2021-11-30

**Authors:** Fadwa Salem Ahmed Al kalali, Essam Mahyoub, Abdulbary Al-Hammadi, Labiba Anam, Yousef Khader

**Affiliations:** 1 Yemen Field Epidemiology Training Program Ministry of Public Health and Population Sana’a Yemen; 2 National Tuberculosis Control Program Ministry of Public Health and Population Sana’a Yemen; 3 Department of Public Health, Community Medicine and Family Medicine Faculty of Medicine Jordan University of Science & Technology Irbid Jordan

**Keywords:** evaluation, surveillance system, tuberculosis, Yemen

## Abstract

**Background:**

Tuberculosis remains a public problem that is considered one of the top causes of morbidity and mortality worldwide. The National Tuberculosis Control Program in Yemen was established in 1970 and included in the national health policy under the leadership of the Ministry of Public Health and Population to monitor tuberculosis control. The surveillance system must be evaluated periodically to produce recommendations for improving performance and usefulness.

**Objective:**

This study aims to assess the usefulness and the performance of the tuberculosis surveillance system attributes and to identify the strengths and weaknesses of the system.

**Methods:**

A quantitative and qualitative evaluation of the national tuberculosis surveillance system was conducted using the Centers for Disease Control and Prevention’s updated guidelines. The study was carried out in 10 districts in Sana’a City. A total of 28 public health facilities providing tuberculosis services for the whole population in their assigned catchment areas were purposively selected. All participants were interviewed based on their involvement with key aspects of tuberculosis surveillance activities.

**Results:**

The tuberculosis surveillance system was found to have an average performance in usefulness (57/80, 71%), flexibility (30/40, 75%), acceptability (174/264, 66%), data quality (4/6, 67%), and positive predictive value (78/107, 73%), and poor performance in simplicity (863/1452, 59%) and stability (15%, 3/20). In addition, the system also had a good performance in sensitivity (78/81, 96%).

**Conclusions:**

The tuberculosis surveillance system was found to be useful. The flexibility, positive predictive value, and data quality were average. Stability and simplicity were poor. The sensitivity was good. The main weaknesses in the tuberculosis surveillance system include a lack of governmental financial support, a paper-based system, and a lack of regular staff training. Developing an electronic system, securing governmental finances, and training the staff on tuberculosis surveillance are strongly recommended to improve the system performance.

## Introduction

Tuberculosis is an infectious disease caused by mycobacterium tuberculosis bacteria [1,2]. It remains a major cause of ill health, one of the top 10 causes of death worldwide, which has taken a higher ranking than HIV/AIDS [3]. According to World Health Organization (WHO) estimates, nearly 10 million people were infected with tuberculosis in 2019 [3]: 5.6 million men, 3.2 million women, and 1.2 million children. There were 1.4 million that died with tuberculosis among HIV-negative people, while 208,000 deaths were among HIV-positive people.

Tuberculosis can affect anyone anywhere, but almost 90% of those who fall sick with tuberculosis have been living in one of the 30 high tuberculosis burden countries. Eight countries account for two-thirds of the total, with India leading the count, followed by Indonesia, China, the Philippines, Pakistan, Nigeria, Bangladesh, and South Africa. The WHO Eastern Mediterranean Region contributes 819,000 (8.5%) of the people who developed the disease, with an estimated incidence rate of 114 per 100,000 populations [4].

Yemen has a moderate tuberculosis burden. According to WHO estimates, 14,000 patients with tuberculosis were reported in 2019, and the incidence rate was estimated as 48 per 100,000 population in 2019 [5]. The surveillance system must be evaluated periodically and produce recommendations for improving performance and usefulness. Therefore, this evaluation aims to assess the usefulness and performance of the tuberculosis surveillance system attributes and to identify the strengths and weaknesses of the system.

## Methods

### Study Design

A quantitative and qualitative evaluation was conducted to assess the performance of the tuberculosis surveillance system using the Centers for Disease Control and Prevention’s (CDC) updated guidelines for Evaluating Public Health Surveillance Systems [6].

### Study Setting and Duration

The study was carried out in 28 tuberculosis sentinel sites for the whole population in their assigned catchment areas in Sana’a City during October 1 to December 31, 2018.

### Study Population

All participants have interviewed based on their involvement with key aspects of tuberculosis surveillance activities: seven managers and one data entry personnel at a central level and one tuberculosis coordinator and one lab supervisor at the governorate level, and at the peripheral level, 10 tuberculosis coordinators from the district level and 11 medical officers and 24 lab technicians from the health facilities.

### Data Collection and Analysis

The National Tuberculosis Control Program (NTCP) documents (strategic plan, guidelines, annual reports, and databases) were reviewed to describe the system. In-depth interviews were used with participants based on their involvement with key aspects of tuberculosis surveillance activities. Verbal consent was obtained from all respondents who participated in the study. Semistructured questionnaires were used to collect information related to surveillance system attributes including flexibility, stability, simplicity, and acceptability at the four levels. The usefulness level was assessed using questions with (yes 1 or no 0) answers, while the other surveillance attributes were assessed using a three-point Likert scale (3 agree, 2 neutral, 1 disagree).

### Scoring System

Specific indicators were used to assess each performance attributes.

The score percent was calculated by the following:




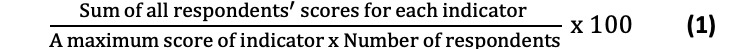




The overall score percent was calculated by the following:




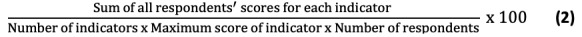




The score percent of each attribute was interpreted as the following: greater than 80% was ranked as good, between 60% to 80% was ranked as average, and lesser than 60% was ranked as poor [7,8]. Data quality was assessed by reviewing documents such as quarter report, while the sensitivity and positive predictive value (PPV) were calculated using the following equation:

Sensitivity = (true positive A / true positive A + false negative C) **(3)**

Predictive value = (true positive A / true positive A + false negative B) **(4)**

The date was analyzed using Excel 2013 (Microsoft Corporation) and Epi Info version 7.2 (CDC) to calculate frequency and percentage.

## Results

### Desk Review Findings

#### Description of the Tuberculosis Surveillance System

The NTCP was established in 1970 in the primary health care sector at the Ministry of Public Health and Population (MoPHP). The Tuberculosis Control Program is a tool for tuberculosis control strategy implementation within a national health system. As such, the NTCP, a vehicle for the directly observed treatment short course (DOTS) strategy since 1995, was used to reach the global targets for detection of at least 70% of cases and treatment success of at least 85%. The NTCP expanded the DOTS strategy gradually to cover all the existing 333 districts until the DOTS coverage in the population reached 100% by the end of December 2007. The MoPHP was established and organized as a central unit of the NTCP in the framework of a national health program in 1995 [9].

As of 2006, the NTCP has adopted the WHO Stop Tuberculosis Strategy and has initiated the development of components. In late 2015, the NTCP adopted the WHO End Tuberculosis Strategy as a national policy to prevent, manage, and control tuberculosis [10]. There are four tuberculosis centers located in major governorates (Sana’a City, Aden, Taiz, and Al Hudaydah). They provided outpatient clinical, radiological, and bacteriological culture services. The laboratory network of tuberculosis control consisted of 271 microscopy laboratories [11].

#### Data Flow of the Tuberculosis Surveillance System

**Figure 1:**
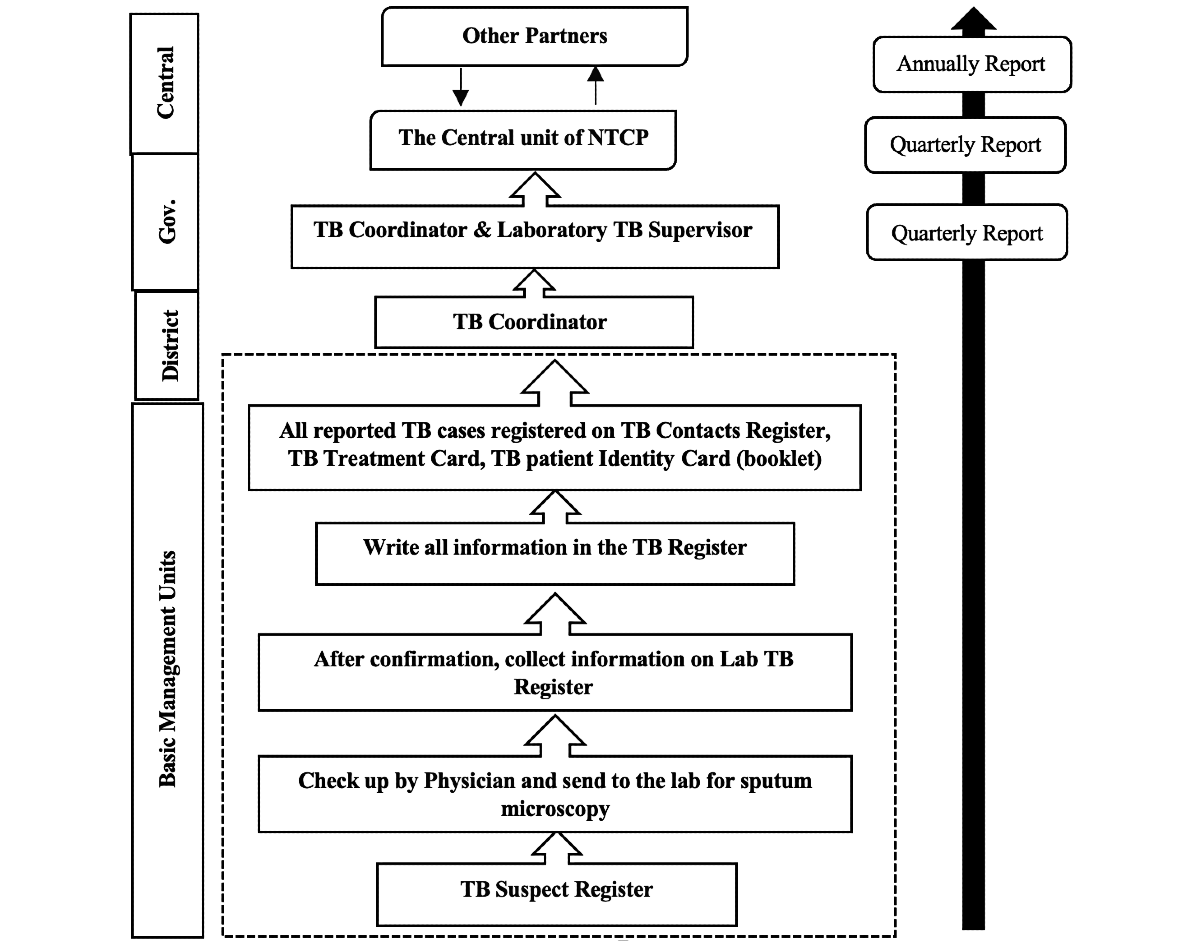
Data flow of the TB Surveillance System in Yemen. Gov: government; NTCP: National Tuberculosis Control Program; TB: tuberculosis.

The following diagram of data flow (Figure 1) describes four levels of responsibility to ensure tuberculosis prevention, care, and control services from peripheral basic management units to the center of the NTCP within the tuberculosis system. Each of these levels has well-defined tasks. The NTCP has 13 forms, registers, and reports. Those were designed for paper-based recording and reporting systems that are used by the surveillance department for management of data at all districts. The tuberculosis control program activities were highly dependent on international donors, the WHO, Japan International Cooperation Agency, and the Global Fund to Fight AIDS.

### Participants’ Characteristics

A total of 54 participants were involved, 57% (n=31) were males and 43% (n=23) females. Almost half of the participants 50% (n=27) were lab technicians.

### Findings From Qualitative Data

#### Usefulness

Table 1 shows that the overall score percent for usefulness was 71% (57/80), indicating an average rank. Only 40% (4/10) of participants mentioned that the tuberculosis surveillance data provided estimates of the tuberculosis magnitude, incidence, prevalence, and mortality, and helped in resource planning, prevention, care, and control.

Some participants mentioned:

The lack of reporting from the private sector leads to the presence of a gap between the estimated number of new cases and the number of actually detected cases.

**Table 1:** The usefulness of the TB surveillance system by score, score percent, and rank in Sana’a City, 2018 (n=10).

Indicators	Score	Score percent	Rank
The system data provide estimates of the TB^a^ magnitude, incidence, prevalence, and mortality	4	40	Poor
The system data were used to monitor trends of TB over time	10	100	Good
The system data can identify areas with anti-TB drugs failure	2	20	Poor
The system data were used for procurement of anti-TB drugs and laboratory reagents when needed	10	100	Good
The system data can be used to recognize the high-risk groups	10	100	Good
The system data helped in resource planning, prevention, care, and control	4	40	Poor
The system data were used to update and develop the national policy strategy for TB control	7	70	Average
The system data were used to assess the impact of interventions	10	100	Good
Overall	57	71	Average

^a^TB: tuberculosis.

### The Tuberculosis Surveillance System Attributes

#### Flexibility

The overall score of flexibility was 75% (30/40) that reveals an average performance. Two out of four indicators of the flexibility attribute, “The system can accommodate any changes in reporting method” and “The system can integrate the surveillance of another disease,” had good rank. The indicator “The system can accommodate data changes with minimum cost and efforts” was poorly ranked (Table 2).

**Table 2:** The flexibility of the tuberculosis surveillance system by score, score percent, and rank in Sana’a City, 2018 (n=10).

Indicators	Score	Score percent	Rank
The system can accommodate with changes in case definition	7	70	Average
The system can accommodate any changes in reporting method	10	100	Good
The system can integrate the surveillance of other disease	10	100	Good
The system can accommodate data changes with minimum cost and efforts	3	30	Poor
Overall	30	75	Average

#### Stability

The overall stability of the system scored 15% (3/20), which indicates a poor performance. Both indicators of stability (“The system is stable after sponsors withdraw their support” and “The system does not require time to manage the data”) had a poor rank (Table 3).

**Table 3:** The stability of the tuberculosis surveillance system by score, score percent, and rank in Sana’a City, 2018 (n=10).

Indicators	Score	Score percent	Rank
The system is stable after sponsors withdraw their support	0	0	Poor
The system does not require time to manage the data	3	30	Poor
Overall	3	15	Poor

#### Simplicity

Regarding simplicity, out of 11 indicators, 6 indicators had a poor ranking. Two of the indicators had a good rank. The overall simplicity of the system scored 59% (863/1452), which indicates a poor performance (Table 4).

**Table 4:** The simplicity and acceptability attributes of the TB surveillance system by score, percent score, and rank in Sana’a City, 2018 (n=44).

Indicators	Score	Score percent	Rank
The system has standard case definitions for TB^a^	121	92	Good
The case definition for TB is easy to use	111	84	Good
Report forms are available	97	73	Average
Report forms are easy to fill	70	53	Poor
Data collection is not time consuming	61	46	Poor
Transmitting data to the central level is easy	59	45	Poor
Follow-up of cases is easy	48	36	Poor
Anti-TB drugs and laboratory reagents are available in a health facility to confirm diagnosis	99	75	Average
Staff received training for TB surveillance	64	48	Poor
Training courses are performed frequently	47	36	Poor
The system is responsive to suggestions	86	65	Average
Overall	863	59	Poor

^a^TB: tuberculosis.

#### Acceptability

Regarding the acceptability attribute, the statement related to willingness to participate in the tuberculosis surveillance system had an average rank of 88% (116/132). However, the satisfaction with the tuberculosis surveillance system had a poor rank of 44% (58/132). The overall acceptability score was 66% (174/264), which indicates average performance (Table 5).

**Table 5:** The acceptability attributes of the TB surveillance system by score, percent score, and rank in Sana’a City, 2018 (n=44).

Indicators	Score	Score percent	Rank
I am willing to participate in the system	116	88	Average
I am satisfied with the TB^a^ surveillance system	58	44	Poor
Overall	174	66	Average

^a^TB: tuberculosis.

### Findings From Quantitative Data

#### Data Quality

Regarding data quality, we have reviewed four tuberculosis reports; each report includes six format types. These formats were compared with the database. Four out of six forms were complete and accurate. However, the completeness and accuracy of the other two forms were zero. The overall data quality score was 67% (4/6), which ranked as average (Table 6).

**Table 6:** The completeness and accuracy of TB reports in Sana’a City, 2018.

Forms	Completeness (%)	Accuracy (%)
TB^a^ case finding	100	100
Demographic characteristics	100	100
Sputum smear microscopy conversion	100	100
TB treatment outcomes	100	100
TB suspect	0	0
TB contacts	0	0
Overall	67	67

^a^TB: tuberculosis.

#### Sensitivity

The sensitivity of the tuberculosis surveillance system was 96%, which ranked as good. The sensitivity calculated by using the formula of (A / A + C) was as follows: sensitivity = 78 / 81 × 100 = 96% (Table 7).

**Table 7:** Distribution of direct smear microscopy and culture of tuberculosis cases to assess the sensitivity and positive predictive value in Sana’a City, 2018

DSM^a^	Culture, n		Total
	Positive	Negative	
Positive	78 (true positive A)	29 (false positive B)	107
Negative	3 (false negative C)	9 (true negative D)	12
Total	81	38	119

^a^DSM: direct smear microscopy.

#### Positive Predicative Value

The PPV of the tuberculosis surveillance system was 73%, which ranked as average. It was calculated by using the formula of (A / A + B), that is, the following: PPV = 78 / 107 × 100 = 73% (Table 7).

### Strengths and Weaknesses of the Tuberculosis Surveillance System

Although the tuberculosis surveillance system had strength points that included the presence of an infrastructure system, qualified human resources, availability of antituberculosis drugs, and the presence of the coordination for tuberculosis activities at all levels, it had many weak points such as depending on external donors and a bureaucrat structure (a large number of hard copies) that lead to delay in its implemented activities in addition to a lack the motivation of human resources, lack of refreshment training for medical officers, poor coordination with other health sectors (private), and high turnover among medical officers at a peripheral level due to political crises and security situations.

## Discussion

### Principal Findings

Evaluation of any surveillance system is the cornerstone for its improvement and ensuring proper morbidity and mortality indicators. It is important to identify the weaknesses and strengths of the system and provide decision makers with evidence-based data to decide on its continuity.

Our study findings showed that the tuberculosis surveillance system was useful. It helps in monitoring trends of tuberculosis and estimates the need for antituberculosis drugs and laboratory reagents at all levels. These findings are similar to other studies in Afghanistan, Yemen, and Pakistan [7,8,12].

The flexibility of the tuberculosis surveillance system was ranked as average. It has been integrated with HIV and AIDS. The NTCP has a counseling clinic where each tuberculosis case was referred to undergo voluntary counseling and testing. Our findings are consistent with the results found in previous evaluations carried out in Pakistan and Zimbabwe [12,13].

Regarding the stability of the tuberculosis surveillance system, it was poor because of its total dependency on the donors in addition to the government’s support for first-line antitubercuolosis drugs and the salary of the staff being completely suspended since 2014, as consequences of political and security crises. In addition, prolonged procedures to release funds from the Global Fund to implement tuberculosis control activities may lead to abandoning the activity implementation. Although, this finding is similar to what has been reported in Yemen and Zimbabwe [8,13]. Other studies showed stable tuberculosis surveillance systems [7,14].

The simplicity of the system was poorly rated for several reasons including data collection being time-consuming, the staff not receiving adequate training on tuberculosis surveillance, training courses not performed frequently, and report forms not easy to fill. Similar findings have been reported in Afghanistan and Zimbabwe [7,13]. However, this finding is not consistent with the findings in previous studies in Yemen, Pakistan, and South Africa [8,12,14].

This evaluation shows that the acceptability was average, as the focal points are willing to participate in the dengue surveillance system; however, they were poorly satisfied. This finding agrees with the findings of others in Afghanistan and Yemen [7,8] but disagrees with other studies in Harare City [15].

The data quality of the tuberculosis system was average. These findings were consistent with findings of other studies conducted in Harare City [15] but inconsistent with another study in the Republic of South Africa [14,16].

The sensitivity and the PPV of the system were good and average, respectively. These results are aline with a previous study in Afghanistan and Eden District [7,14]. However, they disagree with a study in Pakistan [12].

### Limitations

This evaluation was carried out in Sana’a City and targeted only the health facilities providing tuberculosis services (purposive sample) due to time and funds constraints.

### Conclusions

The tuberculosis surveillance system was found useful. Flexibility, PVP, and data quality were average. Stability, acceptability, and simplicity were poor. The sensitivity was good.

The main weaknesses in the tuberculosis surveillance system included a lack of governmental finances, a paper-based system, and a lack of regular staff training. Developing an electronic system, securing governmental financial support, and training the staff on tuberculosis surveillance are strongly recommended to improve the system performance.

## Abbreviations

CDC: Centers for Disease Control and Prevention
DOTS: directly observed treatment short course
MoPHP: Ministry of Public Health and Population
NTCP: National Tuberculosis Control Program
PPV: positive predictive value
WHO: World Health Organization
